# Direct osteosynthesis in the treatment of atlas burst fractures: a systematic review

**DOI:** 10.1186/s13018-024-04571-9

**Published:** 2024-02-08

**Authors:** He-Gang Niu, Jing-Jing Zhang, Yi-Zhu Yan, Kun Yang, Yin-Shun Zhang

**Affiliations:** Department of Orthopedics, The First Affiliated Hospital of Anhui Medical University, No.218 Jixi Road, Hefei, 230022 Anhui Province People’s Republic of China

**Keywords:** Atlas fracture, Osteosynthesis, Transoral, Posterior, Systematic review

## Abstract

**Purpose:**

The treatment of unstable atlas fractures remains a controversial topic. The study aims at assessing the prognosis and efficacy of osteosynthesis for unstable atlas fractures through a review of the current literature and additionally aims to compare outcomes between the transoral and posterior approaches.

**Methods:**

A systematic review of databases including PubMed, EMBASE, Cochrane, Web of Science, CNKI, and Wanfang was conducted. Titles and abstracts were screened by two reviewers to identify studies meeting pre-defined inclusion criteria for comprehensive analysis.

**Results:**

The systematic review included 28 articles, 19 employing the posterior approach and 9 utilizing the transoral approach. It covered osteosynthesis in 297 patients with unstable atlas fractures, comprising 169 treated via the posterior approach and 128 via the transoral approach. Analysis revealed high healing rates and clinical improvement in both approaches, evidenced by improvements in the visual analog scale, range of motion, atlantodens interval, and lateral displacement distance post-surgery.

**Conclusion:**

Osteosynthesis offers effective treatment for unstable atlas fractures. Both transoral and posterior approaches can achieve good clinical outcomes for fracture, and biomechanical studies have confirmed that osteosynthesis can maintain the stability of the occipitocervical region, preserve the motor function of the atlantoaxial and occipito-atlantoaxial joints, and greatly improve the quality of life of patients. However, variations exist in the indications and surgical risks associated with each method, necessitating their selection based on a thorough clinical evaluation of the patient's condition.

## Introduction

Atlas fracture, a significant upper cervical spine injury, constitutes 25% of occipitocervical injuries 2–13% of cervical spine injuries and 1–2% of spinal fractures [1–3]. Primarily resulting from car accidents and falls, these fractures are complicated by the unique anatomy of the area and its proximity to vital centers. Consequently, they often lead to varying degrees of nerve and spinal cord damage, manifesting as occipital and cervical stiffness and pain in mild cases, potentially jeopardizing life safety [4].

Traditionally, the transverse atlantal ligament (TAL) is considered the primary structure ensuring atlantoaxial stability, and its integrity is a crucial factor in evaluating atlas fracture stability [5]. Currently, treatment of atlas burst fracture involving TAL rupture remains controversial. Non-operative approaches often lead to fracture malunion or nonunion, resulting in suboptimal clinical outcome [6]. Therefore, numerous scholars advocate for early surgical treatment [7, 8]. Traditional surgical methods, such as atlantoaxial fusion or occipitocervical fusion [9–11], often involve sacrificing upper cervical motion function [12]. To preserve the mobility of the atlantoaxial joint, many scholars have recently explored and documented various open reduction and internal fixation (ORIF) techniques for unstable atlas fractures. Subsequent clinical follow-ups indicated that patients, including those with TAL injuries, did not exhibit significant atlantoaxial instability post-surgery, and the clinical outcomes were generally positive [13–16]. Biomechanical studies further confirmed that other occipitocervical structures can maintain occipitocervical stability after atlas fracture reduction, even in cases of TAL injury [17].

The existing literature on osteosynthesis for atlas fractures predominantly comprises case reports and retrospective studies, with few systematic reviews. Consequently, we undertook a systematic review of the latest literature, aiming to: (1) evaluate the effectiveness of osteosynthesis in treating atlas fractures, and (2) assess analyze the complications and indications related to the two surgical methods. We aim to provide insights that will assist neurosurgeons and orthopedic surgeons to choose appropriate methods of osteosynthesis techniques for atlas fracture treatment.

## Methods

### Literature search

A systematic assessment was performed in accordance with the Preferred Reporting Items for Systematic Reviews and Meta-Analysis (PRISMA) guidelines to assess the effectiveness of osteosynthesis in the treatment of atlas fracture [18]. This involved a comprehensive search across PubMed, EMBASE, Cochrane, Web of Science, CNKI, and Wanfang to locate relevant articles. These databases were searched up to March 2023. The search strategy encompassed a blend of terms: [(atlas) OR (first cervical vertebra) OR (C1) OR (Jefferson)] AND [(osteosynthesis) OR (reduction) OR (fixation) OR (treatment) OR (fracture) OR (fractures)], utilized as either free-text keywords or Medical Subject Heading (MeSH) terms. The terms ‘‘dan jie duan’’ and ‘‘huan zhui’’ or ‘‘C1’’ used in searches on the CNKI and WanFang databases. We manually examined the reference lists of retrieved articles for additional relevant works, reviewing abstracts for potential full-text analysis and inclusion. Two independent reviewers (H-G.N. and J-J.Z.) conducted the screening and selection of articles. Disagreements were settled via discussion. In cases of unresolved disagreement, a third reviewer (Y–Z.Y.) was tasked with an independent assessment.

### Inclusion and exclusion criteria

After conducting the search, duplicate entries were eliminated. The remaining articles were then screened for relevance based on their titles and abstracts. Articles considered for the full review underwent further evaluation for final inclusion, adhering to the predefined criteria: either an anterior transoral approach or a posterior internal fixation route for osteosynthesis in treating atlas fracture. A second reviewer repeated the screening process to validate the results. Any discrepancies were reconciled. This study encompassed all published prospective, retrospective, case series, and case report studies. Exclusions were made for studies on cadavers, laboratory or animal research, and finite element analysis. Neither meta-analyses nor systematic reviews were considered.

### Data extraction and analysis

The data obtained from the articles were utilized to fill the corresponding fields in the Excel table. Should an article lack the necessary information, the relevant cells in the spreadsheet were labeled as 'Not Available' (NA). Upon selection of the eligible key information was extracted, including the first author's name, publication date, country of origin, study design, patient demographics (age and gender), patient count, surgery duration, follow-up period, bleeding details, type of implant used, and nature of the fracture. The second crucial objective involved assessing the effectiveness of either the anterior transoral approach or posterior internal fixation for atlas fracture. This assessment was based on the comparison of various metrics, including VAS scores, ROM, ADI, LMD, TAL status, infection status, complications, fracture fusion rates, and clinical improvement. Due to the absence of homogeneous randomized or non-randomized comparative studies, conducting a meta-analysis to contrast the two treatment methods was not feasible. Consequently, we undertook a qualitative synthesis of all outcome measure, framing this analysis as a systematic review. The included studies underwent critical assessment, with adjustments made to the level of evidence based on the methodology of previously described prediction studies.

## Results

### Study search

**Fig. 1:**
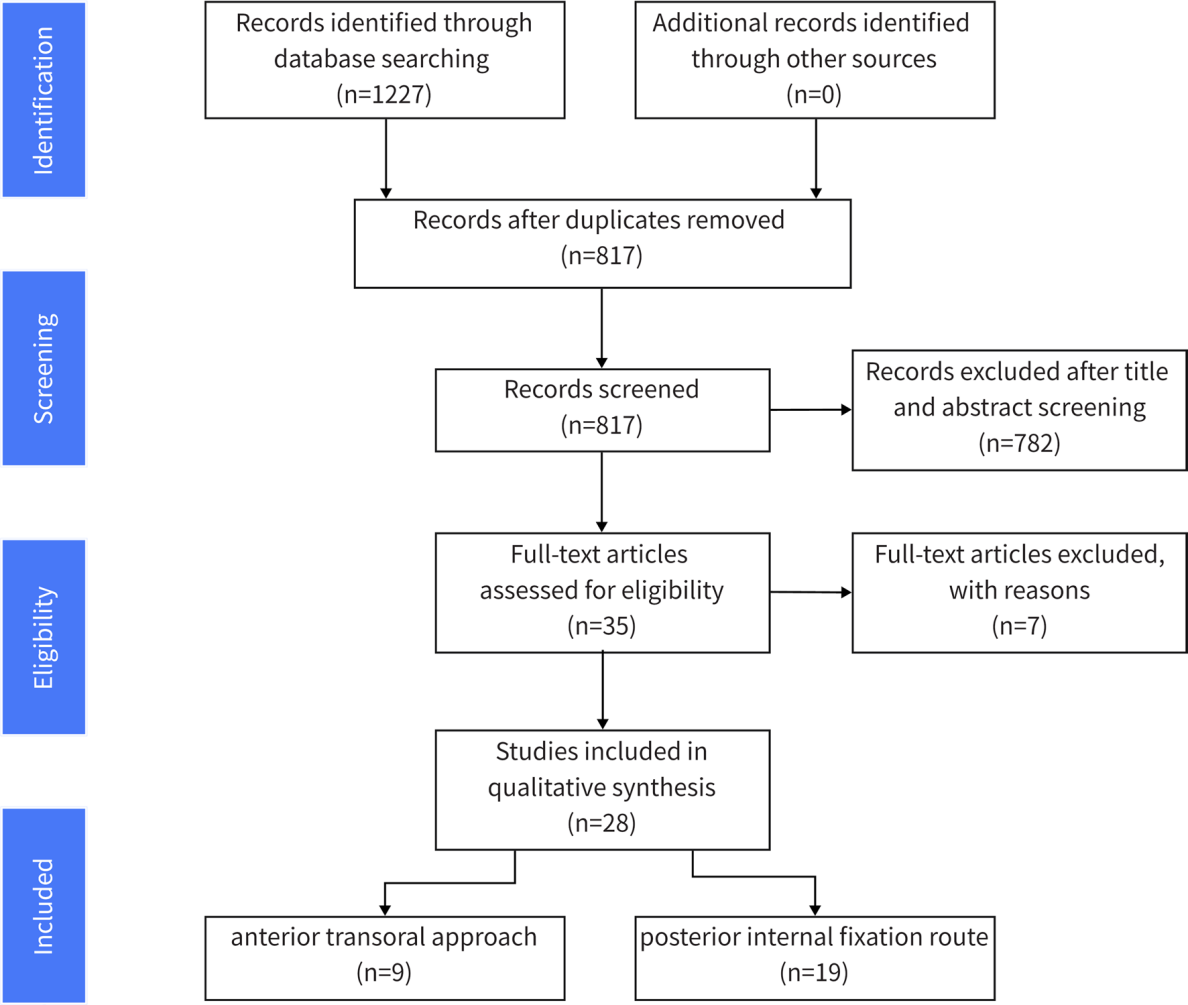
Flowchart of search strategy

A visual summary of the research selection process is depicted in Fig. 1. Initially, the literature search yielded 1227 papers. Following the removal of duplicates, 817 articles remained for subsequent screening. A subsequent examination of titles and abstracts led to the exclusion of 789 studies. Ultimately, 28 articles encompassing 297 patients were included in this systematic review. This included data on 128 patients from 9 articles focusing on the anterior transoral approach, and 169 patients from 19 articles pertaining to the posterior internal fixation route.

### Study and patient characteristics

This experimental study's design comprised 17 retrospective studies, one prospective study, seven case reports, and three case series, with an absence of randomized controlled trials. The articles, published between 2004 and 2023, had study populations varying from 1 to 38 patients. The analysis encompassed 26 single-country studies and 2 international collaborations. Among the single-country studies, 17 originated in Asia, 3 in North America, 4 in Europe, and 2 in Oceania. The international collaborations involved partnerships between China and the United States, as well as Singapore and Australia.

### Implant situation

In posterior internal fixation procedures, the implants utilized included polyaxial screws-rod, monoaxial screws-rod, polyaxial screws-plates, and self-made screws -plates. Among these, polyaxial screws-rods were the most frequently used. Most reported posterior ORIF techniques generally use the implantation of polyaxial pedicle screws in the lateral mass on both sides of C1, which are connected by a single titanium rod and then compressed for reduction and fixation [19–21]. Intraoperative fractures of the posterior atlas arch can be easily reduced under direct vision, and effective internal reduction of the laterally displaced lateral mass can be achieved by compression between the lateral mass screws. However, the fracture of the anterior atlas arch cannot be anatomically reduced due to the tail swing during compression of the polyaxial screws. He et al. employed a self-designed polyaxial screw-plate structure for the posterior C1 ORIF, offering benefits such as a lower profile and reduced C2 nerve root stimulation. However, this method was not entirely effective in reducing anterior arch fractures [14]. Following this, Gumpert et al. [22] and Zhang et al. [7] utilized a monoaxial lateral mass screw-rod system for posterior osteosynthesis of atlas fractures. The convergence of the front of the two monoaxial screws and the compression of the screw end during tightening can effectively reduce the anterior arch fractures, but the reduction system still had significant shortcomings, and the technical requirements were too high to be generalized [23]. Building on this, Yang et al. [23], employing a self-made lateral mass screw-plate system, successfully achieved satisfactory reduction of both the anterior and posterior arches, while also simplifying the surgical procedure.

The transoral approach, including transpharyngeal, transpalatal, transmaxillary, and transmandibular methods, was employed earlier. In 2004, Ruf et al. [24] introduced for the first time a motion-preserving technique known as C1-ring osteosynthesis. This method, utilizing a lateral mass screw-rod structure via a transoral approach, demonstrated significant clinical efficacy in treating unstable atlas fractures. Ma et al. [16] and Hu et al. [15] implemented transoral anterior C1-ring osteosynthesis for unstable atlas fractures using a reconstructed plate, which lacked a repositioning mechanism. Zou et al. [25] utilized a laminoplasty plate for anterior C1-ring osteosynthesis in similar cases. This technique, as reported by Zou et al. [25], effectively reduced not only anterior atlas arch fractures but also lateral mass dislocations, while simultaneously addressing posterior atlas arch fractures. Tu et al. [26] employed a Jefferson-fracture reduction plate for treating atlas burst fractures. This system optimally reduces C1 fractures via an anterior approach, with the inserted plate and screws not impeding midline wound closure and effectively minimizing lateral mass displacement.

### Infection situation

Over the past two decades, the efficacy of transoral route osteosynthesis for atlas burst fractures has been substantiated. Nevertheless, many surgeons remain reluctant to employ this technique due to unfamiliarity with the transoral approach and the theoretical increase in infection risk it poses. Compared to the oral approach, the posterior approach is typically considered to carry a lower risk of infection. However, the only two infected patients in this study underwent the posterior approach, developing superficial postoperative incision infection which resolved following intensive dressing changes. Among the nine transoral route procedures, there were no signs or symptoms of infection observed after surgery or during follow-up. The primary technical challenge currently is that the soft tissue posterior to the pharynx is insufficiently thick to adequately cover the plate or rod, thereby elevating the risk of wound infection [26]. However, the oropharynx is a contaminated site, and preventing oropharyngeal infection is crucial for success. Meticulous preparation before surgery, maintaining sterility during the operation, and appropriate postoperative care can mitigate the risk of pharyngeal wound infection. Additionally, precise suturing of the posterior pharyngeal wall wound to avoid any dead space is another critical strategy [27].

### Radiographic outcomes

Radiological values, comprising LMA, ADI, and TAL, were assessed in 19 studies. Of these, in 10 studies employing the posterior approach, with the exception of the study reported by Zhang et al. [7], only 3 patients exhibited an ADI greater than 4 mm as seen in cervical lateral flexion radiographs at the last follow-up. In the remaining studies, LMD and ADI showed a significant reduction post-ORIF. In the cohort of 109 patients described by TAL, 91 patients evaluated for TAL, 91 experienced TAL injury. In five studies utilizing the anterior approach, postoperative LMD and ADI values were significantly reduced. Conversely, Tu et al. reported three patients exhibiting an ADI greater than 4 mm at the final, yet without neurological symptoms or neck pain [26]. Post-ORIF, reductions in LMD and ADI values were observed in both anterior and posterior approaches.

### Fusion rates

Twenty-six articles assessed the rate of bone fusion post-surgery in patients with atlas fractures (Tables 1, 2). All studies utilizing the anterior approach reported bone fusion rates, with all 9 studies focused on anterior approach treatment of atlas fractures indicating a high fusion rate, at 100%. In contrast, two studies employing the posterior approach did not provide healing rates, but among the remaining 17, 15 achieved a 100% fusion rate. Gelinas-Phaneuf et al. documented eight patients, of whom only six experienced bone healing [28]. Shin et al. observed that computed tomography (CT) images of 9 patients demonstrated bone healing 6 months after surgery [29]. CT scans of 11 patients, taken 1 year after surgery, revealed complete bone union, resulting in a healing rate of 92% [29].

**Table 1:** Report of posterior internal fixation approach osteosynthesis for atlas fracture

Study/publication year	Study design	Level of evidence	Country	Age (years)	Number of patients	Gender (M/F)	Surgical time (min)/Follow-up (mo)	Blood loss (mL)	Implant	Fracture type
Li et al. [21]	Case report	IV	China	33	2	1/1	NA/12	NA	Screws + rod	Jefferson fracture
Jo et al. [30]/2011	Case report	IV	Korea	31	1	1/0	NA/8	NA	Screws + rod	Jefferson fracture
Abeloos et al. [20]	Case report	IV	Belgium	25	1	1/0	NA/7	NA	Polyaxial screws + rod	Landells: type II
Bransford et al. [31]	Case series	IV	USA	54.3	3	3/0	NA/14.5	NA	Polyaxial screws + rod	NA
Hu et al. [19]	Retro study	II	China	35.6(20–60)	12	8/4	70.5/22	150(100–300)	Polyaxial screws + rod	Landells: type II
He et al. [14]	Retro study	II	China	43.5(23–68)	22	16/6	86(68–122)/22.56 ± 18.0(12–32)	120(90–400)	Polyaxial screws + plate	NA
Shatsky et al. [13]	Retro study	II	USA	43 (21–86)	12	9/3	NA/17	NA	Polyaxial screws + rod	Landells: type II
Gumpert et al. [22]	Case series	IV	Australia	50.7	3	2/1	NA/NA	NA	Monoaxial screws + rod	Landells: type II (1); type III (2)
Ottenbacher et al. [32]	Case report	IV	Australia	70	1	1/0	NA/NA	NA	Polyaxial screws + rod	Jefferson fracture
Gelinas-Phaneuf et al. [28]	Retro study	II	USA	37.9(20–71)	8	6/2	NA/12.6(1–49)	NA	Polyaxial screws + rod Monoaxial screws + rod	NA
Zhang et al. [7]	Retro study	II	China	50.3	9	6/3	127/17.4	106	Monoaxial screws + rod	Landells: type II (7); type III (2)
Kumar et al. [33]	Case report	IV	Singapore, Australia	39	1	1/0	NA/12	NA	Screws + rod	NA
Li et al. [34]	Retro study	II	China	36.7(18–57)	25	19/6	76(80–120)/41(6–72)	150(100–300)	Screws + plate	NA
Rajasekaran et al. [35]	Prospe study	II	India	39.6	5	NA	77 ± 13.96/40.8(25–59)	84.4 ± 8.04	Polyaxial screws + rod	NA
Gao et al. [36]	Retro study	II	China	38.5 ± 11.3	23	15/8	93.6 ± 28.1/15.3 ± 8.5	158.5 ± 53.6	Monoaxial screws + rod	Landells: type II
Shin et al. [29]	Retro study	II	Korea	37 (21–63)	12	8/4	111.92 ± 7.14/12	125 ± 78.33	Polyaxial screws + rod	4-part atlas fracture: 3 lateral mass fractures: 9
Ottenbacher et al. [37]	Case series	IV	Germany	60.8	5	4/1	116.8/11.6	480	NA	NA
Bao et al. [38]	Retro study	II	China	51.8	14	10/4	82.1 ± 8.8/21.0 ± 6.4	84.3 ± 16.8	Screws + rod	NA
Yang et al. [23]	Retro study	II	China	47.0 ± 9.7	10	9/1	108.7 ± 20.1/16.7 ± 9.6	98.0 ± 41.3	Screws + plate	Landells: type II (7); type III (3)

Study	VAS(preoperative-last follow-up)	ROM(°)	ADI(mm)	LMD(preoperative-postoperative)(mm)	TAL injury	infection rate, n (%)	Complications	Fusion (bone consolidation/ healing)/Clinical improvement, n (%)
Li et al. [21]	NA	NA	NA	NA	NA	NA	NA	2(100)/NA
Jo et al. [30]/2011	NA	NA	NA	NA	0(0)	NA	NA	1(100)/1(100)
Abeloos et al. [20]	NA	NA	NA	NA	NA	NA	NA	1(100)/NA
Bransford et al. [31]	NA	NA	NA	NA	0(0)	NA	Suboptimally placed right C1 lateral mass screw:1(33)	3(100)/NA
Hu et al. [19]	7.52 ± 3.2(6–10) to 1.80 ± 2.12(0–4)	36(27–41)flexion; 40(30–49)extension; 32(16–38)left bending;29(14–37) right bending;62(36–75) rotation of C1–C2	2.4(2.0–3.0)flexion;1.6(1.0–2.0)extension	7.5 (5–13) to 3.0(1–6.0)	5(42)	NA	Partial breach of the pedicle screw:1(8.3);screw displacement:1(8.3)	12(100)/NA
He et al. [14]	7.26 ± 1.4(4–8) to 1.96 ± 1.1(0–3)	Axial:42.4 ± 11.0(28.6–63.5)	NA	NA	22(100)	NA	NO	22(100)/22(100)
Shatsky et al. [13]	0.7 ± 1.6 at last follow-up	NA	ADI ≤ 4 at last follow-up	7.1 to 2.1	11(92)	NO	Errant lateral mass screw placement: 1 (8.3); Occipital-C1 arthrosis:1 (8.3)	12(100)/NA
Gumpert et al. [22]	NA	45–90 left rotation; 45–70 right rotation	NA	NA	1(33)	NA	Screw penetrated into the spinal canal:1(33)	3(100)/NA
Ottenbacher et al. [32]	NA	38/0/32 degree rotation of the head	less than 3 mm	NA	1(100)	NA	NA	NA/NA
Gelinas-Phaneuf et al. [28]	5.1 to 0.8	Two patients had decreased ROM	NA	NA	NA	NO	Transient neurological deficit due to vertebral artery dissection:1(13) Lucency around a screw:1(13)	6(75)/7(88)
Zhang et al. [7]	1.0 ± 0.87(0–2)	All the patients had a well-preserved ROM	3.2;more than 4 mm:3(33)	7.0 ± 2.2 to restored completely	8(89)	NO	NO	9(100)/NA
Kumar et al. [33]	NA	NA	NA	NA	NA	NA	NA	1(100)/1(100)
Li et al. [34]	6.8 ± 1.5 to 1.6 ± 0.2	NA	NA	4.35 ± 2.03 to 1.35 ± 1.13	NA	NA	Screw breaks through pedicle bone cortex:1(4)	NA/NA
Rajasekaran et al. [35]	NA	35.4 flexion;43.8 extension;24.8 left bending;28.4 right bending;71.6 left rotation;73.6 right rotation	NA	14.6 ± 1.34 to 5.2 ± 1.64	NA	NA	NO	5(100)/5(100)
Gao et al. [36]	7.2 ± 1.8 to 1.5 ± 1.1	NA	NA	7.3 ± 2.1 to 0.7 ± 0.5	18(78)	2(9)	NA	23(100)/NA
Shin et al. [29]	0.92 ± 0.99 (0–3)	36.17 ± 5.42 (27–41) flexion;40 ± 5.79 (30–45)extension;75.83 ± 3.59 (70–80) rotation	3.79 ± 1.56 postoperative extension;3.13 ± 1.01 postoperative flexion;3.42 ± 1.34 postoperative 6 months;3.33 ± 1.24 postoperative 1 year	NA	12(100)	NO	NA	11(92)/NA
Ottenbacher et al. [37]	NA	NA	2.25 flexion;1.5 extension	5.16 to 2.14 right; 5.76 to 3.02 left	5(100)	NA	NO	5(100)/NA
Bao et al. [38]	6.5 ± 1.3 to 1.9 ± 0.8	79.7 ± 6.0 (70–88) flexion and extension; 82.1 ± 2.8 (76–85) left/right bending; 143.5 ± 8.4 (129–155) left/right rotation	NA	NA	NA	NO	NO	5(100)/NA
Yang et al. [23]	0.6 ± 0.7 at last follow-up	All patients preserved almost full ROM	2.3 ± 0.8 at last follow-up	7.1 ± 1.9 to restored completely	8(80)	NO	NO	10(100)/10(100)

Retro retrospective, Prospe prospective, M/F male/female, NA not available, VAS visual analog scale, ROM range of motion, ADI atlantodens interval, LMD lateral mass displacement, TAL transverse atlantal ligament

**Table 2:** Report of anterior transoral approach osteosynthesis for atlas fracture

Study/publication year	Study design	Level of evidence	Country	Age (years)	Number of Patients	Gender (M/F)	Surgical time (min)/Follow-up (mo)	Blood Loss (mL)	Implant	Fracture Type
Ruf et al.[24]	Retro study	II	Germany	28(17–37)	6	NA	160(80–245)/77(21–165)	425(50–700)	Polyaxial screws + rod	NA
Hu et al.[27]	Case report	IV	China	20	1	1/0	NA/14	NA	Screws + plate	NA
Yin et al.[39]	Retro study	II	China	48(26–67)	15	10/5	NA/18(8–77)	NA	Screws + plate	NA
Ma et al.[16]	Retro study	IV	China	47.7 ± 13.9	20	12/8	101.4 ± 12.9(76–124)/48.5 ± 20(12–81)	NA	Screws + plate	NA
Hu et al.[15]	Retro study	II	China, USA	33(23–62)	12	8/4	100(80–120)/16(12–28)	300(100–500)	Screws + plate	Landells: type I(3);type II(5); type III (2)
Keskil et al.[40]	Case report	IV	Turkey	51	1	1/0	125/NA	NA	Screws + wire	NA
Li et al.[4]	Retro study	II	China	45.8(20–74)	38	27/11	65(58–90)/38(6–70)	86(40–150)	Screws + plate	NA
Zou et al.[25]	Retro study	II	China	47.8(32–66)	13	6/7	86.9 ± 16.7(60–120)/17.4 ± 4.4(12–24)	52.3 ± 16.9(30–80)	Screws + plate	Landells: type II
Tu et al.[26]	Retro study	II	China	32–67	22	12/10	181.3 ± 185.00/26.84 ± 10.23	144.21 ± 155.67	Screws + plate	Gehweiler: type I(7); type III (15)

Study	VAS(preoperative-last follow-up )	ROM(°)	ADI(mm)	LMD(preoperative-postoperative)(mm)	TAL injury	Infection rate, n (%)	Complications	Fusion (bone consolidation/ healing)/clinical improvement, n (%)
Ruf et al.[24]	NA	38(30–40)flexion;45(30–50)extension;35(15–45)left bending;37(25–45)right bending;60(30–80)left rotation;66(40–80)right rotation;39.2(10–61)rotation(MRI)	3.6(3–5); 1.6(1–2)extension;	Preoperative: 13.5(8–19) Postoperative: 4.3(1–8) Follow-up: 5.2(0–12)	NA	NA	NO	6(100)/NA
Hu et al.[27]	NA	NA	NA	NA	NA	NA	NA	1(100)/NA
Yin et al.[39]	NA	NA	NA	NA	NA	NA	NO	15(100)/NA
Ma et al.[16]	Preoperative: 6.0 ± 1.3(4–8) Postoperative: 1.3 ± 1.0	39.0 ± 12.0:axial	NA	NA	NA	NO	NO	20(100)/NA
Hu et al.[15]	NA	35(28–40) flexion; 42(30–48)extension; 30 left bending; 28 right bending; 50(35–72) rotation	2.5(2.0–3.0)flexion; 1.5(1.0–2.0)extension	7.0 (5–12) to 3.5 (1–6.5)	NA	NA	NA	12(100)/12(100)
Keskil et al.[40]	NA	NA	NA	NA	NA	NA	NA	1(100)/NA
Li et al.[4]	6.8 ± 1.3 to 1.2 ± 0.2	NA	Preoperative:2.82 ± 0.78 last follow-up:2.38 ± 0.41	Preoperative:5.12 ± 2.83 last follow-up:1.34 ± 1.13	NA	NO	NO	38(100)/NA
Zou et al.[25]	Preoperative: 6.9 ± 0.9 Postoperative: 0.4 ± 0.7	49.3 ± 10.8:axial ROM at last follow-up	NA	6.1 ± 1.5 to 1.0 ± 0.6	NA	NO	NO	13(100)/13(100)
Tu et al.[26]	Preoperative: 7.42 ± 3.92 Postoperative: 2.17 ± 1.33 Follow-up: 0.28 ± 0.13	NA	ADI > 4 at last follow-up:3(14)	Preoperative: 7.13 ± 1.46 Postoperative: 1.02 ± 0.65 Follow-up: 0.53 ± 0.21	9(41)	NO	atlantoaxial dislocation:3(14)	22(100)/NA

### Clinical outcomes

Eight articles reported clinical improvement following osteosynthesis for atlas fractures: two articles focusing on the transoral approach and five articles on the posterior approach showing significant clinical improvement in all patients post-surgery. Additionally, one article addressing the posterior approach mentioned a patient who was lost to follow-up [28]. Fourteen articles observed improvements in postoperative VAS pain scores, indicating a mean VAS score of less than 2 for both the anterior and posterior approaches. Likewise, fourteen articles detailed postoperative ROM in patients, 13 reported that patients regained ROM postoperatively, whereas Gelinas-Phaneuf et al. recorded two patients with persistent neck pain and decreased ROM during recent follow-ups [28].

### Complications

Seven studies documented complications, with six attributed to the posterior approach, potentially reflecting a scarcity of research on the anterior approach. The majority of these complications included incorrect placement of the lateral mass screw, the partial breach of the pedicle screw, screw insertion into the vertebral canal, transient neurological deficits from vertebral artery dissection, screw penetration through pedicle bone cortex, and the atlantoaxial dislocation.

### Discussion

Atlas fractures are commonly caused by injuries from car accidents, heavy objects, or falls. The force exerted on the skull transmits progressively downward through the cranial-occipital condyles axis to the atlas. Due to their unique wedge-shaped structure, the lateral masses enable strong axial force to convert into horizontal outward stress. This results in the separation of the lateral mass from the fragile area at the junction of the anterior and posterior atlas arches, and the lateral displacement of the lateral mass on both sides. This mechanism is typically the cause of atlas burst fractures. There are several classification systems for atlas fractures, the three most widely utilized are the Jefferson classification [41], the Landells et al. classification [42], and the Gehweiler et al. classification [43].

The integrity of the TAL is crucial in evaluating the stability of an atlas fracture. While the TAL is a key stabilizing structure, the bony structures of the occipito-atlanto-axial complex, the joint capsule, other transverse ligaments, and the longitudinal ligaments also significantly contribute to atlas stability. Therefore, Dickman et al. [44] concluded that even in half ring fracture of atlas with intact TAL, where the TAL prevents separation and displacement of the lateral mass, the fracture may still exhibit rotatory displacement around the ligament's attachment point, constituting an unstable atlas fracture. The stability of an atlas fracture cannot be solely determined based on the integrity of the TAL alone. Lee et al. [45] conducted a review and analysis of numerous cases of atlas fractures and concluded that only a solitary anterior arch fracture or a simple posterior arch fracture of the atlas with complete TAL constitutes a stable fracture, whereas all other types of atlas fractures are considered unstable.

For atlas burst fractures, a range of treatment options and fixation methods exist, which remain subject to debate. Historically, non-operative treatment was the predominant choice for managing atlas burst fractures. However, this approach often resulted in poor fracture reduction. With advancements in surgical techniques, surgical treatment has increasingly become the preferred method [46]. Traditional atlantoaxial or occipitocervical fusion for atlas burst fractures results in the sacrifice of ROM in the upper cervical spine, substantially diminishing the patient's postoperative quality of life [9]. Recently, ORIF has been advocated as a treatment for atlas burst fractures, aiming to minimize surgical trauma while preserving ROM in the upper cervical spine. Currently, there is debate regarding the treatment of unstable atlas fractures associated with TAL rupture in ORIF, particularly concerning the potential for atlantoaxial instability post-surgery due to TAL rupture. Clinical follow-ups have not identified significant atlantoaxial instability in patients with TAL injuries. Additionally, biomechanical studies have confirmed that the burst fracture of the atlas results from vertical trauma. Even when the TAL of the atlas is damaged, other stable occipital and cervical structures merely lose tension due to skull sinking after displacement of the atlas's lateral mass to both sides. Upon reduction of the atlas fracture, this axial tension can be restored, thereby re-establishing the main stable structure of the occipitocervical region [21, 47]. Despite the irreparability of the TAL, the stability of the occipitocervical region remains intact under physiological stress.

The treatment of unstable atlas burst fractures with osteosynthesis is reported to involve combined anterior–posterior approach internal fixation, an anterior transoral approach, and posterior screw rod or plate reduction and internal fixation. Bohm et al. [48] addressed the issue of anterior arch reduction by supplementing posterior atlas fracture compression internal fixation with anterior surgery, employing wires to tighten the heads of lateral screws on both sides penetrating the anterior cortical bone. However, this procedure is more invasive and challenging, hindering its widespread adoption.

Posterior fixation relies on the technique of implanting atlas lateral mass screws and pedicle screws. The technology for atlas lateral mass screw implantation is now relatively advanced, offering strong screw fixation and ample surgical space during posterior surgery. However, posterior fixation often leads to incomplete reduction of anterior arch fractures of the atlas due to tail swing during compression with polyaxial screws. Gumpert et al. [22] and Zhang et al. [7] addressed this issue by employing monoaxial screws. However, the presently employed posterior monoaxial screw-rod system exhibits a high internal fixation notch. Additionally, due to the angled U-shaped slot of the monoaxial screw relative to the transverse connecting rod, if the screw fails to maintain pressure during locking, it may shift to both ends of the connecting rod. Furthermore, controlling the rotation of the transverse connecting rod can also be challenging [23]. Therefore, the operation is intrinsically complicated, potentially resulting in postoperative challenges such as impaired reduction of paravertebral muscles, chronic bursitis, and chronic neck pain [23]. Recently, Yang et al. [23] demonstrated that effective repositioning of both anterior and posterior arches could be accomplished using a self-made lateral mass screw-plate system through a simpler procedure.

In contrast to cases with an intact atlas ring, the insertion of lateral mass screws in atlas burst fractures poses greater difficulty, primarily due to the displacement and instability of the lateral mass [7]. This increases the risk of complications, including incorrect placement of lateral mass screws, insertion of screws into the vertebral canal, and screws penetrating through the pedicle bone cortex. Therefore, extensive clinical experience is essential for the insertion of lateral mass screws in atlas burst fractures. Utilizing a high-speed power drill to create the screw path and employing computer-assisted navigation techniques prior to the insertion may enhance the procedure's success. During posterior surgery, careful dissection is conducted to expose the posterior arch of the atlas. Cottonoids are employed to protect the C1-C2 venous plexus, while bipolar cautery and Gelfoam are used for effective control epidural venous bleeding [19, 21].

Due to the deep location, limited space, and restricted field of vision inherent in the transoral approach, the risk of screw implantation is elevated for atlas fractures with fracture lines near the lateral mass, potentially resulting in surgical failure. Additionally, this approach carries drawbacks such as a high rate of postoperative infection and challenging technique, leading to a postoperative complications is as high as 75% [15, 24]. Wound infection and dehiscence are frequent complications, representing approximately 9–22% of cases [49, 50]. Ma et al. [16] and Hu et al. [15] suggest that infection rates can be diminished or even averted with proper preoperative preparation and postoperative care. Instances of cerebrospinal fluid leakage, meningitis, neurological impairment, and pseudomeningocele have also been documented [51]. Approximately 4% of patients experience breathing, swallowing, and speech difficulties, necessitating gastrostomy and tracheostomy in some cases [52]. The primary issue with the existing transoral approach technique is that the soft tissue behind the pharynx lacks sufficient thickness to adequately cover the plate or rod, subsequently elevating the risk of wound complications [26]. Moreover, achieving an effective reduction of atlas fractures in deep and narrow spaces is challenging. Consequently, the use of specialized devices or spinal implants is recommended in anterior approach surgery for atlas burst fractures. This approach not only minimizes the complications associated with the anterior method but also offers benefits such as direct reduction of anterior arch fractures, enhanced healing, and the absence of visible scars post-surgery.

### Strength and limitations

This is the first systematic review to describe the prognosis and clinical efficacy of anterior and posterior osteosynthesis for the treatment of atlas fractures. We have offered a thorough and systematic analysis from multiple perspectives and dimensions. The paper encompasses studies in English and other languages, including interventions common in various regions. An exhaustive literature search across relevant databases was performed, employing an extensive search strategy to guarantee thorough inclusion of all pertinent data.

Studies addressing anterior treatment of atlas fractures might be underrepresented in the literature, potentially leading to an underestimation of the infection risks associated with anterior surgery. Moreover, as most included studies were case series, the evidence level is considered low. No definitive randomized trials have been conducted, and it is improbable that such trials will be conducted given the topic's specificity. The retrospective nature and research design of these studies could introduce potential biases in the results and conclusions. Due to differences in fracture classification and the use of different implants, there may be errors in the analysis of these two approaches. Lastly, a meta-analysis was not performed as part of this systematic review due to the heterogeneity of reported outcomes and limitations in data strength and sample size.

## Conclusion

In summary, both the transoral and the posterior approaches are feasible for osteosynthesis in treating atlas burst fractures. The two surgical approaches are similar in treatment effect, both of which can reconstruct the stability of the atlas, preserve the ROM of the upper cervical spine to the maximum extent, and greatly improve the postoperative quality of life of the patients. They are effective for treating atlas burst fractures and merit wider adoption. The optimal choice between the two approaches should consider fracture type, patient age and needs, surgeon's experience, implant choice, surgical contraindications, and particularly patient-specific factors in the elderly.

## Data Availability

The data generated or analyzed in this study are included in the published article.

## Abbreviations

VAS: Visual analog scale
ROM: Range of motion
ADI: Atlantodens interval
LMD: Lateral displacement distance
TAL: Transverse atlantal ligament
ORIF: Open reduction and internal fixation
PRISMA: Preferred reporting items for systematic reviews and meta-analysis
MeSH: Medical Subject Heading
CT: Computed tomography
